# Gellan gum formulations containing natural polyphenolic compounds to treat oral candidiasis

**DOI:** 10.1128/spectrum.00798-25

**Published:** 2025-08-06

**Authors:** Narchonai Ganesan, Lewis Oscar Felix, Biswajit Mishra, Liyang Zhang, Charilaos Dellis, Fadi Shehadeh, Danielle Wu, Lissette A. Cruz, R. M. Arce, Eleftherios Mylonakis

**Affiliations:** 1 Department of Medicine, Houston Methodist Research Institute and Houston Methodist Hospital 167626 , Houston, Texas, USA; 2 Department of Pharmaceutical Chemistry, National and Kapodistrian University of Athens 68994, Panepistimioupolis Zografou, Athens, Greece; 3 Department of Electrical and Computer Engineering, National Technical University of Athens 414915 , Athens, Greece; 4 Department of Diagnostic and Biomedical Sciences, The University of Texas Health Science Center at Houston School of Dentistry 67334, Houston, Texas, USA; 5 Department of Bioengineering, Rice University 3990, Houston, Texas, USA; 6 Center for Craniofacial Research Instrumentation Core, The University of Texas Health Science Center at Houston School of Dentistry 67334, Houston, Texas, USA; 7 Department of Periodontics and Oral Hygiene, The University of Texas Health Science Center at Houston School of Dentistry 67334, Houston, Texas, USA; University of Debrecen, Debrecen, Hungary

**Keywords:** biofilm, caffeic acid phenethyl ester, *Candida albicans*, ellagic acid, hyphae, artificial chewing simulation

## Abstract

Oral candidiasis (OC) is caused by *Candida albicans*, targeting immunocompromised individuals and developing drug resistance, highlighting the need for advanced therapeutics. Polyphenols such as caffeic acid phenethyl ester (CAPE) and ellagic acid (EA) display antifungal and immune-modulating properties. Incorporating CAPE and EA in gellan gum (GG) formulation enhances their applicability and effectiveness against OC. We developed GG-based formulations loaded with CAPE (1,000 µg/mL), EA (1,000 µg/mL), or CAPE + EA (1,000 µg/mL each) against *C. albicans*. GG formulations containing gellan (0.6% and 1.0%), genipin (5 mM), polyethylene glycol 400 (0.5%), and sorbitan monooleate 80 (0.5%) demonstrated enhanced release of CAPE and EA. The 0.6% GG formulation reduced *C. albicans* CFU by 2–6 log_10_ within 30 min (*P* < 0.05) and biofilm mass by 48% (CAPE, *P* = 0.0034), 60.7% (EA, *P* = 0.0980), and 70% (CAPE + EA, *P* = 0.0181). Both 0.6% and 1.0% GG formulations inhibited hyphae (*P* < 0.0001). GG formulations showed high viability of human red blood cells (92%–94%) and human gingival cells (61%–69%). In artificial chewing simulations (ACS), 0.6% GG exhibited 67.7% (30 min), 55.9% (60 min), and 35.8% (120 min) for CAPE release, and 48.2% (30 min), 45.1% (60 min), and 42.1% (120 min) for EA. In 1% GG, about 44.07% (30 min), 43.8% (60 min), and 29.5% (120 min) of CAPE and 55.8% (30 min), 49.6% (60 min), and 50.6% (120 min) of EA were released. The present study is the first to evaluate the efficacy of CAPE- and EA-loaded GG formulations against *C. albicans* under ACS, thereby supporting their potential development for OC treatment.

**IMPORTANCE:**

*Candida albicans* causes OC and presents challenges due to rising antifungal resistance and recurrence in immunocompromised patients. Existing antifungal treatments for OC often fail due to limited bioavailability, short retention times, adverse side effects, and the bitter taste of formulations, impacting patient adherence. CAPE and EA are recognized for their antifungal and immunomodulatory properties but face practical limitations in therapeutic applications, such as poor bioavailability and stability. The present study addresses these challenges by developing GG-based formulations incorporating CAPE and EA. The formulation exhibited significant antifungal efficacy against *C. albicans* biofilms and hyphal formation, reducing fungal viability under simulated mechanical chewing conditions. These GG-based systems showed minimal cytotoxicity, indicating promising biocompatibility and suitability for oral application. Therefore, the study presents the first report of CAPE/EA-loaded GG formulations under simulated chewing that highlights the importance of innovative therapeutic strategies to improve clinical outcomes in OC treatment.

## INTRODUCTION

Oral candidiasis (OC) is a fungal infection of the oral mucous membranes caused by the *Candida* sp., especially *Candida albicans* (1). Predisposing factors for OC include poor oral hygiene, smoking, malnutrition, extremes of age, excessive antifungal use, and immunosuppressive conditions like human immunodeficiency virus (HIV)/acquired immunodeficiency syndrome (AIDS) (2, 3). OC is a common oral infection in HIV patients, often an early HIV indicator, with *C. albicans* prevalence ranging from 0.9% to 83.0% in patients with HIV (4, 5). In elderly and immunocompromised individuals, dentures often serve as reservoirs for *C. albicans*, leading to persistent reinfection and complicating the management of OC (6). These challenges have necessitated the use of various conventional treatments, including antifungal agents such as nystatin, miconazole, and fluconazole (7). However, prolonged use of conventional antifungal medications can increase the risk of OC because high or improper doses can disrupt the balance of natural flora in the oral cavity, causing overgrowth of *Candida* spp. (8). These limitations emphasize the need to develop new therapeutic approaches.

The use of natural molecules derived from biological sources has the potential to replace conventional antifungal agents. Among the various existing natural compounds, polyphenols are known for their protective properties on human health, including antioxidant, anti-inflammatory, anticancer, and antimicrobial effects (9–11). Caffeic acid phenethyl ester (CAPE) and ellagic acid (EA) are polyphenol-rich, natural molecules that have been studied for their anticandidal properties (12). EA is abundant in various herbs, fruits, and vegetables, including raspberries, strawberries, and walnuts (13), while CAPE is found in plants and is extracted from propolis, a resinous substance produced by honeybees (14). EA exists in the form of hydrolyzed tannins, known as ellagitannins, which exhibit antioxidant, antifungal, and anti-inflammatory activities (15). Similarly, CAPE has demonstrated anti-inflammatory (16), antioxidant (17), immunomodulatory (18), and antimicrobial effects (19). Furthermore, CAPE has shown synergistic effects with caspofungin, fluconazole, and nystatin (20, 21). Previous reports have demonstrated anticandidal activity for both CAPE and EA against *C. albicans* and *Candida auris* (12, 22, 23).

Despite their promising biological activities, both CAPE and EA have poor aqueous solubility and low bioavailability, significantly limiting their clinical potential (12). CAPE is highly susceptible to enzymatic hydrolysis by plasma esterases, resulting in rapid systemic clearance and a short biological half-life (24–26). Wang et al. (27) reported a half-life of just 0.35 h for CAPE at a concentration of 5 µg/mL at 37°C, highlighting its instability under physiological conditions. The poor water solubility of CAPE also restricts its pharmacological efficacy in both *in vitro* and *in vivo* environments (28). Similarly, EA exhibits limited solubility, poor membrane permeability, and rapid metabolic conversion that contributes to its low systemic bioavailability (29). Direct supplementation of CAPE and EA remains suboptimal due to their physicochemical limitations. Therefore, encapsulating CAPE and EA using biocompatible polymers will enhance solubility and control the release (30). For instance, Garcia et al. (22) formulated CAPE within gellan gum (GG), a mucoadhesive polysaccharide approved by the Food and Drug Administration (FDA), which offers excellent thermal and pH stability, tunable gelation, and protection against enzymatic degradation and salivary washout. Similarly, EA has also been encapsulated in cyclodextrins and nanoparticles to improve its solubility and release kinetics, enhancing its therapeutic utility (31).

Developing a more patient-friendly alternative, such as a palatable formulation in the form of a gel or gummies, may potentially enhance treatment compliance and improve clinical outcomes. Among the biomaterials used for the formulation of natural compounds, GG has shown promising applications in local, targeted, nasal, and oral delivery systems (32, 33). GG is a water-soluble anionic exopolysaccharide produced by the bacterial genus *Sphingomonas*, which gels in the presence of cations (34). Its polymer chain consists of a tetrasaccharide repeating unit of L-rhamnose, D-glucose, and D-glucuronate. GG has an average molecular weight of approximately 500 kDa and can tolerate heat and acid stress during fabrication (35). Notably, GG is approved by the FDA as a gelling agent in food (36). Recently, GG has gained attention in the pharmaceutical and medical industries due to its functional and material properties. These include stability after heating or pH variations, dispersibility, and a versatile texture that allows for the adjustment of hardness, firmness, brittleness, and elasticity (37). GG is also environmentally friendly, sustainable, and inexpensive (38–40). Additionally, studies have shown that carrier systems using GG exhibit good biocompatibility with oral tissues and protect incorporated drugs from enzymatic activity in saliva (41–43).

Despite their individually potent activity at low minimum inhibitory concentration (MIC) levels (12), to our knowledge, no previous study has co-loaded CAPE and EA into a single GG-based formulation or evaluated their combined antifungal efficacy against *C. albicans*. Based on the benefits of GG, CAPE, and EA, we focused this study on fabricating a GG formulation that allows slow and efficient release of CAPE and EA to kill *C. albicans* in its planktonic, hyphal, and biofilm forms under *in vitro* and mechanically simulated conditions.

## MATERIALS AND METHODS

### Chemicals

CAPE (Tocris Bioscience, Bristol, UK) and EA (Sigma-Aldrich Co., St. Louis, MO, USA) were used in this study and dissolved in absolute ethanol (99% purity) at a concentration of 10 mg/mL. Calcium chloride (CaCl_2_), genipin, 1× phosphate-buffered saline (PBS) (Gibco), yeast peptone dextrose (YPD), Roswell Park Memorial Institute Medium (RPMI) 1640, fetal bovine serum (FBS), Dulbecco’s Modified Eagle Medium (DMEM), and artificial saliva (Pickering Laboratories, California, USA) were purchased from Fisher Scientific (Massachusetts, USA). Polyethylene glycol (PEG) 400, Sorbitan Monooleate (Span) 80, and gellan were obtained from Sigma-Aldrich.

### Fungal strains and culture conditions

*C. albicans* ATCC 18804 was purchased from the American Type Culture Collection (ATCC), and *C. albicans* MLR62 (GFP-expressing wild type) was obtained from the Division of Infectious Diseases, Massachusetts General Hospital, Boston, MA, USA. Both *C. albicans* strains were cultured in YPD for 24 h at 37°C.

### Growth inhibition assay

We grew overnight cultures of the *C. albicans* ATCC 18804 strain in the YPD medium. After incubation, we harvested the cells by centrifuging at 2,700 × *g* for 5 min, washed them twice with 1× PBS, and counted them using an automated cell counter (Luna II; Logos Biosystems, Gyeonggi-do, South Korea). We adjusted the final inoculum concentration to 1 × 10³ cells/mL in YPD medium. Next, we prepared serial dilutions of CAPE (32, 16, 8, 4, and 0 µg/mL), EA (8, 4, 2, 1, and 0 µg/mL), and CAPE + EA (32 + 4, 16 + 2, 8 + 1, 4 + 0.5, and 0 µg/mL) in YPD medium at a final volume of 50 µL. The selected concentrations of CAPE and EA were based on preliminary experiments (date not shown) and supported by previously reported MICs (12) and solubility limits of the polyphenols. Then, we added 50 µL of *C. albicans* inoculum to each sample. Using a microplate reader (SpectraMax M2 Multi-Mode Microplate Reader; Molecular Devices, California, USA), we measured the sample optical density (OD) at 600 nm at 30 min intervals from 0 to 840 min.

### Preparation of CAPE, EA, and CAPE + EAGG formulations

We dissolved 0.6% (wt/vol) and 1.0% (wt/vol) gellan in 100 mL of distilled water at 85°C–90°C for 10 min. Once fully dissolved, we added 0.5% PEG 400 and stirred the solution at 75°C for 10 min. Next, we introduced 0.5% Span 80 and continued stirring at 75°C for another 10 min. Then, we lowered the temperature to 45°C–55°C and gradually incorporated 1,000 µg/mL of CAPE, EA, or CAPE + EA. CAPE and EA did not exhibit any color changes after being dissolved in dimethyl sulfoxide and ethanol or after being incorporated into the GG formulation. We then added 5 mM genipin and stirred for 10 min at 45°C to ensure uniform incorporation. We transferred 100 µL aliquots of each formulation into 300 µL plastic molds and allowed them to set at 4°C for 2 h. We prepared the blank GG formulation using the same procedure as the CAP- and EA-loaded GG formulation, excluding the addition of CAPE and EA. We used the blank GG formulation to assess any potential interference or background effects of the polymer matrix. Table 1 provides the optimized combination of chemical components for improved GG formulation.

**TABLE 1:** Optimization of GG formulation to improve the release efficacy of CAPE and EA^*a*^

Composition	F-1	F-2	F-3	F-4	F-5	F-6	F-7	F-8
Gellan gum (%)	0.6	0.6	0.6	0.6	0.6	1.0	2.0	3.0
Genipin (mM)	5	5	5	–^*b*^	–	5	5	5
PEG 400 (%)	0.5	–	–	0.5	0.5	0.5	0.5	0.5
Span 80 (%)	0.5	0.5	–	–	0.5	0.5	0.5	0.5
CaCl_2_ (mM)	–		–	–	1.0	–	–	–

^*a*^ F, formulation.

^*b*^ –, not added.

To evaluate drug release, we dispensed 100 µL of the respective GG formulations (containing 1,000 µg/mL of CAPE, EA, or CAPE + EA) into a 24-well plate, followed by 900 µL of artificial saliva. We incubated the samples at room temperature with shaking (100 rpm) for 1 h. After incubation, we transferred 100 µL aliquots to UV-transparent, 96-well plates (CLS3635, Corning) and measured absorbance at 250–500 nm at 1 nm intervals using SpectraMax M2 Multi-Mode Microplate Reader (Molecular Devices). We selected the formulations with the highest peak concentration for further experiments.

### Drug-release kinetics

We measured the UV-vis absorption spectra of CAPE, EA, CAPE-GG, and EA-GG formulations to confirm the incorporation of CAPE and EA into GG and to establish a calibration curve for release kinetics. Then, we prepared CAPE-GG and EA-GG formulations at concentrations ranging from 1,000 to 15.26 µg/mL. We added 100 µL of each formulation to 900 µL of artificial saliva in a 24-well plate and incubated the plate at room temperature for 1 h. We used the blank GG formulation as a control to assess any potential interference or background effects of the polymer matrix. Additionally, the potential interference from individual GG formulation components such as PEG 400 (0.5%), Span 80 (0.5%), and genipin (5 mM) was independently evaluated. After incubation, we transferred 100 μL samples to UV-transparent 96-well plates and measured absorbance at 250–500 nm at 1 nm intervals using a spectrophotometer (Molecular Devices). We repeated this procedure for GG, CAPE-GG, and EA-GG formulations. Finally, we measured CAPE and EA alone to compare the rate of drug release and calculated the percentage of drug release using the following formula: drug release (%) = absorbance of drug with GG − absorbance of GG alone at 0 min × 100/absorbance of drug without GG.

### Short-term killing kinetics of the GG formulations under static condition

Briefly, we inoculated *C. albicans* ATCC 18804 planktonic cells (10^5^ cells/mL) in 9 mL artificial saliva and added 1 mL of GG formulations (1,000 µg/mL of CAPE, EA, or CAPE + EA). Fluconazole (1,000 µg/mL)-loaded GG formulation was used as a positive control. The samples were incubated for 1 h, and 100 µL samples were collected every 10 min over the 1 h duration. We serially diluted each sample in artificial saliva and spot plated on YPD agar, and incubated the plates at 37°C for 24 h. After incubation, we counted colony-forming units (CFU) to determine fungal viability (CFU/mL) (44).

### Killing kinetics of the GG formulations over a longer duration under shaking condition

We inoculated C*. albicans* ATCC 18804 planktonic cells (10³ cells/mL) in 9 mL of artificial saliva and added 1 mL of the respective GG formulations (1,000 µg/mL of CAPE, EA, or CAPE + EA). Using an orbital shaker, we incubated the samples at room temperature under shaking at 100 rpm for 4 h. We collected 100 µL aliquots at 0, 30, 60, 120, and 240 min. We serially diluted each sample in artificial saliva, plated them on YPD agar, and incubated them at 37°C for 24 h. After incubation, we counted CFUs to determine fungal viability. We defined fungicidal activity as a ≥3 log_10_ (99.9%) reduction in CFU count from the starting inoculum and fungistatic activity as a <99.9% reduction in growth (44).

### Disruption of initial biofilm formation

In each well of a 24-well plate, we added 1 mL of RPMI medium supplemented with 5% FBS. We inoculated each well with 100 μL of *C. albicans* (10^5^ cells/mL) overnight culture. We incubated the plates at 37°C for 4 h. Afterward, we added 100 µL of the respective GG formulations (CAPE-GG, EA-GG, CAPE + EA GG, or blank-GG) and incubated the samples at 37°C for another 4 h. After the second incubation, we removed the GG formulations and free-floating planktonic cells, washed the wells twice with PBS, and applied 0.4% crystal violet (CV) for 15 min to stain the biofilm biomasses. We washed off excess CV with PBS and destained the biofilm using 70% ethanol (45). We quantified the destained ethanol at 570 nm using a spectrophotometer (Molecular Devices) and calculated the percentage of biofilm disruption using the following formula: biofilm disruption (%) = control/treatment × 100.

### Hypha inhibition

In each well of a 24-well plate, we inoculated 1 mL of RPMI medium supplemented with 5% human serum with an overnight culture of *C. albicans* MLR62 at 0.2 OD at 600 nm. We incubated plates for 4 h at 37°C to allow hyphal formation. After incubation, we added 100 µL of the respective GG formulations (CAPE-GG, EA-GG, CAPE + EA GG, or blank-GG) and incubated the plates at 37°C for another 4 h. We discarded the excess medium and washed the wells twice with PBS (19). Images were acquired using a BZ-X810 fluorescence microscope (KEYENCE, Itasca, IL, USA) at ×40 magnification. Furthermore, the number of hyphae-forming cells was manually counted in three different regions on the slide at ×40 magnification.

### Hemolysis assay

In a 24-well plate, we added 900 µL of 2% human red blood cells (hRBCs) (Rockland Immunochemicals, Pennsylvania, USA) suspended in PBS to each well. Then, we added 100 μL of the respective GG formulations (CAPE-GG, EA-GG, CAPE + EA GG, or blank-GG) to each well. We used Triton X-100 (2%) as a positive control. The plate was incubated at 37°C for 1 h. After incubation, we transferred 100 µL samples to a fresh 96-well plate and centrifuged at 500 × *g* for 5 min. The supernatant fractions were transferred to a fresh 96-well plate, and absorbance was measured at 540 nm using a spectrophotometer (19).

### Cytotoxicity assay using a human gingival fibroblast cell line

We performed a cytotoxicity assay of CAPE-GG, EA-GG, CAPE + EA GG, or blank-GG (22). We cultured human gingival fibroblast-1 (HGF-1) (ATCC, VA, USA) cells in DMEM supplemented with 10% heat-inactivated FBS (Invitrogen, New York, USA) and incubated at 37°C with 5% CO_2_. We seeded 4 × 10⁴ HGF-1 cells per well into 96-well plates and incubated them for 24–48 h to achieve >50% adherence, as previously described (46). Before treatment, we assessed cell morphology and confluence using an inverted phase-contrast microscope (Nikon Eclipse, Tokyo, Japan). To further confirm adherence and viability, we performed cell counting using an automated cell counter (Luna II, Anyang, Gyeonggi-do, South Korea) after trypan blue staining, which confirmed >50% viable and adherent HGF-1 cells. Following adherence, we treated the cells with 0.6% and 1.0% GG formulations loaded with 1,000 µg/mL of CAPE, EA, or a combination of CAPE and EA. Blank GG alone was used as a negative control. A control group of non-treated cells was included. Triton X-100 (2%) was used as a positive control.

We also tested the cytotoxicity of other components in the GG formulation, including PEG 400 (0.5%), Span 80, and genipin (5  mM). We incubated the plates for 24 h, and 4 h before the end of the incubation period, we added 10 µL of 2-(4-iodophenyl)-3-(4-nitrophenyl)-5-(2,4-disulfophenyl)-2H-tetrazolium (WST-1) solution (Roche, Mannheim, Germany) to each well. We monitored WST-1 reduction using a spectrophotometer at 450 nm (Molecular Devices). Assays were performed in triplicate, and cell survival was calculated as a percentage. The lethal dose, LD_50_, was considered the concentration of drug needed to kill 50% of the cells (47).

### Artificial simulative chewing model for the GG formulations

We used the Flexcell FX-5000 Compression System (Flexcell International Corporation, North Carolina, USA) to simulate the mechanical forces of mastication and assess drug release from 0.6% to 1.0% GG formulations, including CAPE-GG, EA-GG, CAPE + EA GG, and blank-GG. The compression system delivers precisely controlled cyclic compression, enabling physiologically relevant simulation of chewing stresses encountered by oral drug delivery systems (48). By applying this model, we evaluated how mechanical stress influences the release kinetics of polyphenols from the hydrogel matrix, mimicking real-world oral cavity dynamics (48).

We used sterile six-well Bioflex BioPress culture plates (BP-3000U) with silicone elastomer well bottoms (Flexcell International Corporation). Before each experiment, the platens were cleaned, autoclaved, and stored in sealed sterilization bags. In each well of a BioPress plate, we added 300 µL of the respective GG formulations into the foam sample holder and adjusted the thread height of the platen above the sample to ensure complete compression and chewing of the sample during the experiment. Each chamber was filled with 3 mL of sterile artificial saliva. We then placed the assembled setup on a compression baseplate and checked for any air leakage or plate imbalance.

We simulated chewing of the GG formulations at room temperature under static compression using the following parameters: shape, 1/2 SINE; minimum, 1; maximum, 10; frequency, 2.0 Hz; duty cycle, 50%; cycles, 1,800; and duration, 0, 30, 60, and 120 min. Afterward, we collected 100  µL samples of artificial saliva at each time point. The aliquots were filtered using 0.22  µm syringe filters (MilliporeSigma, St. Louis, MO, USA) and transferred to 96-well UV-transparent plates for UV-Vis spectroscopic analysis. We measured absorbance at 250–500 nm at 1 nm intervals using a spectrophotometer (Molecular Devices) to determine the release of CAPE, EA, and CAPE + EA.

We also added 10^5^ CFU/mL of *C. albicans* ATCC 18804 to the artificial saliva containing GG formulations in the six-well BioPress culture plates. We collected 100 µL samples at 30 and 60 min time points. Then we serially diluted each sample in artificial saliva, plated them on YPD agar, and incubated them at 37°C for 24 h. After incubation, we counted the CFU per milliliter to determine fungal viability.

### Statistical analysis

All experiments were performed in triplicate (*n* = 3) unless otherwise stated. Data are presented as mean ± SD. Statistical comparisons between control and treatment groups were conducted using two-way analysis of variance followed by Dunnett’s multiple comparisons test. A *P* value of <0.05 was considered statistically significant. All statistical analyses were performed using GraphPad Prism version 10.4.2 (GraphPad Software, San Diego, CA, USA).

## RESULTS

### Growth curve of *C. albicans* in the presence of CAPE, EA, and CAPE + EA

**Fig 1:**
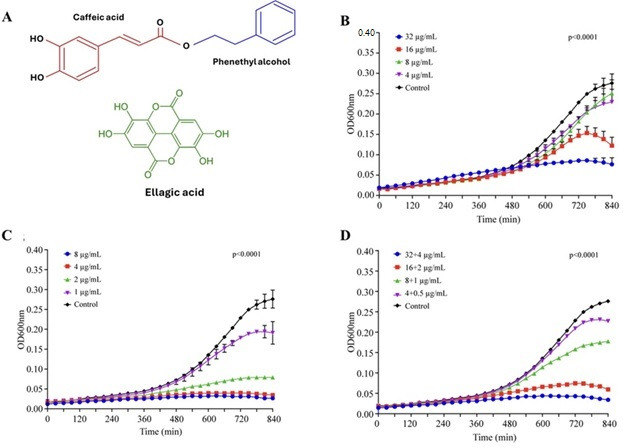
Growth inhibition of *C. albicans* by CAPE, EA, and CAPE + EA (0–840 min). (**A**) Structure of CAPE and EA. (**B**) CAPE (32, 16, 8, 4, and 0 µg/mL) showed maximum inhibition at a concentration of 32 µg/mL. (**C**) EA (8, 4, 2, 1, and 0 µg/mL) demonstrated maximum inhibition at concentrations ranging from 4 to 8 μg/mL. (**D**) CAPE + EA (32 + 4, 16 + 2, 8 + 1, 4 + 0.5, and 0 µg/mL) exhibited maximum inhibition at the concentrations of 32 + 4 and 16 + 2 µg/mL. Data are presented as mean ± SD (*n* = 3).

We conducted a growth curve experiment using various concentrations of CAPE, EA, and CAPE + EA to determine their efficacy against the growth of *C. albicans*. The growth curve experiment determined the minimum concentrations of CAPE, EA, and CAPE + EA required to inhibit *C. albicans* growth within 14 h. CAPE at 32 µg/mL (Fig. 1B) and EA at 4 µg/mL (Fig. 1C) demonstrated maximum inhibition of *C. albicans* growth (Fig. 1B through D). In contrast, concentrations below 16 µg/mL for CAPE and 2 µg/mL for EA showed no inhibitory or killing activities against *C. albicans*. The combination of CAPE + EA (32 + 4 µg/mL and 16 + 2 µg/mL) also inhibited *C. albicans* growth (Fig. 1D).

### Synergistic effect between CAPE and EA against *C. albicans*

We performed a standard checkerboard assay to evaluate the interaction between CAPE and EA against *C. albicans* and calculated the fractional inhibitory concentration index (FICI). The MICs for the individual compounds were 16  µg/mL for CAPE and 2  µg/mL for EA, whereas the MICs in combination were reduced to 2  µg/mL for CAPE and 0.125  µg/mL for EA. Based on the MIC values, the calculated FICI was 0.25, which indicates synergistic interaction between CAPE and EA (Fig. S1).

### Optimization of the GG formulation

**Fig 2:**
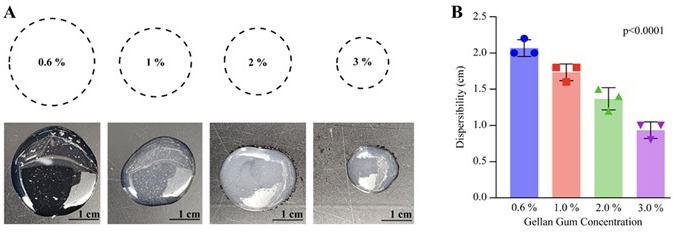
Dispersion of GG at various concentrations. (A) Visual representation of GG dispersion at concentrations of 0.6%, 1.0%, 2.0%, and 3.0% of gellan. (B) Quantitative measurement of GG dispersibility (diameter in cm) shows that the higher concentrations of gellan led to reduced dispersion: 0.6% (2.06 cm), 1.0% (1.73 cm), 2.0% (1.36 cm), and 3.0% (0.93 cm). Data are presented as mean ± SD (n = 3). Statistical differences were analyzed by one-way analysis of variance.

We evaluated the drug-release efficacy of various combinations of GG with PEG 400, Span 80, genipin, and CaCl_2_. Formulation 1, composed of GG (0.6%), genipin (5 mM), PEG 400 (0.5%), and Span 80 (0.5%) loaded with 1,000 µg/mL of CAPE, EA, or CAPE + EA, demonstrated higher drug release than all other concentrations. Within 60 min in saliva, Formulation 1 released CAPE and EA more effectively than other combinations (Fig. S2). Increasing the concentration of GG (0.6%, 1.0%, 2.0%, and 3.0%) reduced its dispersibility (Fig. 2), which could be due to the formulation’s sponge-like texture, causing slower drug release.

### Drug release of CAPE and EA

**Fig 3:**
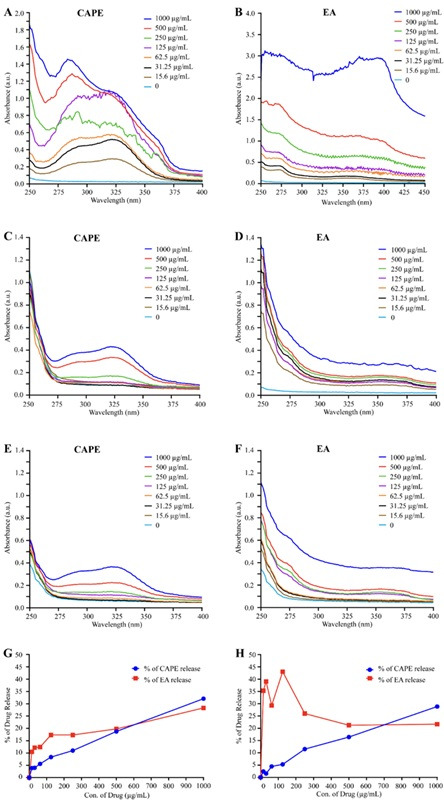
UV-Vis absorption spectra (A–F) and drug release (%) (G and H) of the 0.6% and 1% GG formulations loaded with 1,000 µg/mL of CAPE, EA, or CAPE + EA over 60 min. (A–B) The UV-Vis spectra of varying concentrations (1,000, 500, 250, 125, 75, 31.25, 15.6, and 0 µg/mL) of CAPE and EA were recorded, revealing primary absorption peaks at 325 and 255 nm for CAPE and EA, respectively. (C and D) The UV-Vis absorption spectra of the 0.6% GG formulation loaded with CAPE or EA at 1,000, 500, 250, 125, 75, 31.25, 15.6, and 0 µg/mL concentrations demonstrated a decrease in absorbance as drug concentration decreased over 60 min. (E and F) Similarly, the UV-Vis absorption spectra of the 1% GG formulation loaded with CAPE or EA (1,000, 500, 250, 125, 75, 31.25, 15.6, and 0 µg/mL) exhibited a decrease in absorbance with declining drug concentration at 60 min. (G and H) The percentage of drug release at 60 min was quantified for the 0.6% and 1.0% GG formulations. The maximum drug release of CAPE and EA from the 0.6% GG formulation was 32.07% and 28.3%, respectively. In comparison, the maximum drug release of CAPE and EA from the 1% GG formulation was 28.8% and 43.04%, respectively.

We further evaluated drug release for the GG formulations loaded with varying concentrations (1,000, 500, 250, 125, 62.5, 31.25, and 15.6 µg/mL) of CAPE and EA (Fig. 3A through H) at 60 min. The CAPE-GG and EA-GG formulations exhibited the highest peak concentration at 1,000 µg/mL. In contrast, no significant peaks were detected at the lower concentrations of 125.0, 62.5, 31.25, and 15.6 µg/mL (Fig. 3C through F).

The use of a higher concentration (1,000 µg/mL) in the drug-release kinetics, compared to the lower concentrations (up to 32 µg/mL for CAPE and 8 µg/mL for EA) used in antifungal growth assays, was intentional and based on the technical limitations of the detection method. Specifically, when we loaded CAPE (32 µg/mL) and EA (8 µg/mL) into the GG formulation, the cumulative drug release from the hydrogel matrix remained below the detection threshold of our UV-Vis spectrophotometer, rendering quantification of the release unreliable. Furthermore, as shown in Fig. 3A and B, CAPE and EA at concentrations below 125 µg/mL in solution alone produced absorbance values that were too low for consistent detection and analysis. Therefore, to enable accurate release profiling and to evaluate cytotoxicity under conditions where the released drug could be reliably measured, we used a higher initial loading concentration of 1,000  µg/mL in the drug release, killing kinetics, and cytotoxicity assays.

The 0.6% GG formulations yielded a maximum overall release of 32.07% for CAPE and 28.3% for EA at 60 min. The 1% GG formulation achieved a maximum drug release of 28.8% for CAPE and 43.04% for EA within the same time frame (Fig. 3G through H). These results indicate that under static incubation conditions, both the 0.6% and 1.0% GG formulations facilitated a slow and sustained release of CAPE. However, the 1% EA-GG formulation displayed a sudden burst release of EA ranging from 15.6 to 125.0 µg/mL. No spectral interference was detected from PEG 400 (0.5%), Span 80 (0.5%), or genipin (5 mM) that overlapped with the absorbance spectra of CAPE and EA (Fig. S3).

### Anticandidal activity of the GG formulations within 60 min of treatment

**Fig 4:**
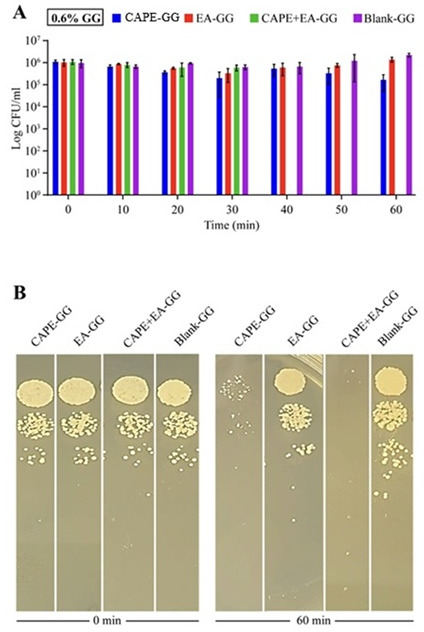
Killing kinetics of *C. albicans* treated with the 0.6% GG formulation loaded with CAPE (1,000 µg/mL), EA (1,000 µg/mL), or CAPE (1,000 µg/mL) + EA (1,000 µg/mL) over 60 min. (A) Quantitative analysis of the anticandidal activity of the 0.6% GG formulations showed significant reductions in viable fungal colonies. Treatment with CAPE (1,000 µg/mL), EA (1,000 µg/mL), or CAPE (1,000 µg/mL) + EA (1,000 µg/mL) resulted in reductions of 6 log₁₀ colony-forming units (CFU) (P = 0.0219), 1.2 log_10_ CFU (P = 0.0184), and 0.8 log_10_ CFU (P = 0.1249), respectively, within 60 min. (B) Visual representation of microbial colonies demonstrated a reduction in CFUs as the duration of treatment increased over 0–60 min. Decreased colony formation indicated effective killing of *C. albicans*. Data are presented as mean ± SD (n = 3). Statistical differences were analyzed by one-way analysis of variance.

To test the effects of the 0.6% and 1.0% GG formulations on planktonic *C. albicans* cultures in artificial saliva, we performed a static killing assay over 60 min. The 0.6% GG formulations containing 1,000 µg/mL of CAPE, EA, or CAPE + EA did not significantly reduce fungal viability within the first 30 min. However, after 40 and higher time points of 60 min, the CAPE + EA GG formulation eliminated all viable fungal colonies, achieving a 6 log reduction in CFUs (*P* = 0.0219). By 60 min, the CAPE-GG formulation reduced fungal viability by 1.2 CFU (*P* = 0.0184), whereas the EA-GG formulation achieved a 0.8 log reduction in CFUs (Fig. 4A and B). Fluconazole (0.6% GG)-treated *C. albicans* exhibited a 2.6 log_10_ CFU/mL increase in growth at 60 min compared to the 0 min time point (Fig. S4).

**Fig 5:**
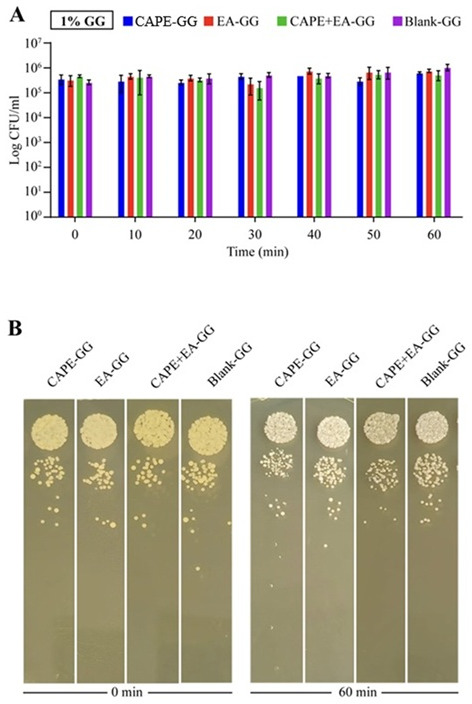
Killing kinetics of *C. albicans* treated with 1% gellan gum (GG) formulations. GG formulation (1%) was loaded with CAPE (1,000 µg/mL), EA (1,000 µg/mL), or CAPE (1,000 µg/mL) + EA (1,000 µg/mL). *C. albicans* were treated over 60 min. (**A**) Quantitative analysis of the anticandidal activity of the 1% GG formulations for CAPE + EA GG (*P* = 0.1649), CAPE-GG (*P* = 0.2413), and EA-GG (*P* = 0.4156) when compared with the blank-GG control at 60 min. (**B**) Visual representation of microbial colonies demonstrating reduced CFUs as over 0–60 min of treatment. Data are presented as mean ± SD (*n* = 3). Statistical differences were analyzed by one-way analysis of variance.

In contrast, the 1% GG formulations containing 1,000 µg/mL of CAPE, EA, or CAPE + EA did not significantly reduce *C. albicans* viability by the 60 min time point. At this time point, the CAPE-GG, EA-GG, and CAPE + EA GG formulations resulted in reductions of 0.4, 0.3, and 0.7 CFU, respectively (Fig. 5A and B). Additionally, killing kinetics experiments using GG formulations loaded with 500 and 250 µg/mL concentrations of CAPE, EA, or CAPE + EA did not show any detectable reduction in *C. albicans* viability after 60 min (data not shown).

### Killing kinetics of the GG formulations against *C. albicans* over a 4 h duration under constant shaking

**Fig 6:**
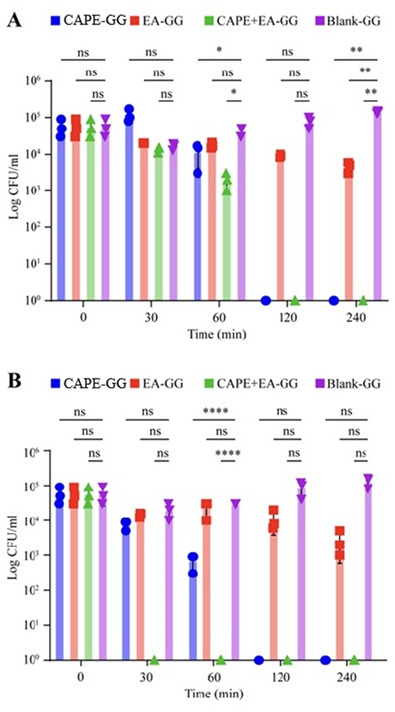
Killing kinetics of *C. albicans* treated with the 0.6% and 1% GG formulations of CAPE (1,000 µg/mL), EA (1,000 µg/mL), or CAPE (1,000 µg/mL) +EA (1,000 µg/mL) over 240 min. (**A**) The 0.6% GG formulation loaded with CAPE (1,000 µg/mL), EA (1,000 µg/mL), or CAPE (1,000 µg/mL) +EA (1,000 µg/mL) showed time-dependent reductions in viable *C. albicans* cells, with CAPE + EA demonstrating the most significant antifungal activity (*P* < 0.05). (**B**) The 1% GG formulation loaded with CAPE (1,000 µg/mL), EA (1,000 µg/mL), or CAPE (1,000 µg/mL) +EA (1,000 µg/mL) showed a similar trend, with CAPE + EA exhibiting enhanced antifungal activity compared with the individual treatments. The higher GG concentration (1%) resulted in slightly slower drug release but sustained antifungal effects over the duration. Data are presented as mean ± SD (*n* = 3). Statistical differences were analyzed by one-way analysis of variance. *P  <  0.05, **P <  0.005, ****P  <  0.0001. ns, non-significant.

Then, we conducted a killing kinetics assay to evaluate the release of CAPE and EA from 0.6% and 1.0% GG formulations under continuous shaking conditions over a prolonged period (60–240 min). We used different inoculum densities to align with the specific objectives and kinetics of each assay. For the short-term killing assay, a higher inoculum (10⁵ CFU/mL) was employed to detect rapid antifungal effects within a limited time frame (≤1 h), ensuring sufficient fungal biomass for accurate CFU enumeration during frequent sampling. In contrast, the 4 h extended killing assay used a lower inoculum (10³ CFU/mL) to prevent the growth of *C. albicans* during prolonged incubation, allowing for a more precise assessment of time-dependent fungicidal activity. Continuous shaking enhances drug release from the GG formulation, which in turn accelerates the killing of *C. albicans*. At 60 min, the CAPE-GG, EA-GG, and CAPE + EA GG formulations reduced *C. albicans* viability by 0.3, 0.2, and 1.2 log_10_ CFU, respectively. Treatment for 120 and 240 min with the CAPE-GG and CAPE + EA GG formulations resulted in a 5 log reduction in CFUs (no viable colonies), whereas the EA-GG formulation achieved a maximum reduction of 1.5 log_10_ CFU at 240 min (Fig. 6A).

In contrast, the 1% CAPE + EA GG formulation decreases *C. albicans* viability at 30 min. The 1% CAPE-GG and EA-GG formulations reduced *C. albicans* viability by 0.3 and 0.1 log_10_ CFU, respectively, whereas the CAPE + EA GG formulation resulted in a 5 log_10_ CFU reduction (no viable colonies) at 30 min (Fig. 6B). *C. albicans* viability continued to decrease throughout treatment. The CAPE + EA GG formulation consistently reduced C*. albicans* viability at 60, 120, and 240 min. Furthermore, no viable colonies were detected with the CAPE-GG formulation treatment at 120 and 240 min (no viable colonies). The EA-GG formulation reduced *C. albicans* viability by 1.0 and 2.3 log_10_ CFU at 10 and 240 min, respectively.

### Disruption of *C. albicans* initial biofilm formation by the GG formulations

**Fig 7:**
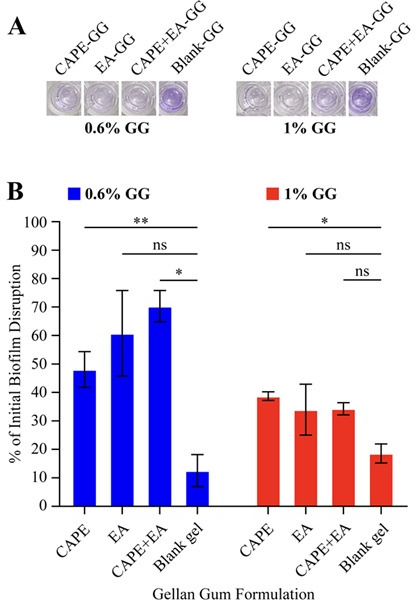
Biofilm disruption of *C. albicans* using the GG formulations loaded with CAPE (1,000 µg/mL), EA (1,000 µg/mL), or CAPE (1,000 µg/mL) + EA (1,000 µg/mL). (**A**) Qualitative analysis of biofilm disruption using crystal violet staining. The 0.6% and 1% GG formulations with CAPE (1,000 µg/mL), EA (1,000 µg/mL), or CAPE (1,000 µg/mL) + EA (1,000 µg/mL) caused reductions in crystal violet, indicating *C. albicans* initial biofilm disruption. (**B**) Bar graph of the percentages of initial biofilm disruption after treatment with the 0.6% and 1.0% GG formulations loaded with CAPE (1,000 µg/mL), EA (1,000 µg/mL), or CAPE (1,000 µg/mL) + EA (1,000 µg/mL). Blank-GG formulations without drugs were used as controls. The 0.6% GG formulation disrupted 48.05% (CAPE-GG), 60.75% (EA-GG), and 70.27% (CAPE + EA GG) of *C. albicans* biofilm. The 1% GG formulation disrupted 38.6% (CAPE-GG), 33.9% (EA-GG), and 18.5% (CAPE + EA GG) of *C. albicans* biofilm. Data are presented as mean ± SD (*n* = 3). Statistical differences were analyzed by one-way analysis of variance. *P  <  0.05, **P <  0.005. ns, non-significant.

Next, to assess *C. albicans* biofilm disruption efficacy of GG formulation, we tested the effect of CAPE, EA, and CAPE + EA on disrupting the initial biofilm formation of *C. albicans* under static conditions. The 0.6% GG formulations (CAPE-GG, EA-GG, and CAPE + EA GG) exhibited 48.05%, 60.75%, and 70.27% disruption of *C. albicans* biofilm, respectively (Fig. 7A) compared with a 9% disruption in the control blank-GG. The 1% GG formulations (CAPE-GG, EA-GG, CAPE + EA GG, and blank-GG) disrupted 38.6%, 33.9%, 34.2%, and 18.5% of *C. albicans* biofilm, respectively (Fig. 7B).

### Inhibition of *C. albicans* hyphal formation by the GG formulations

**Fig 8:**
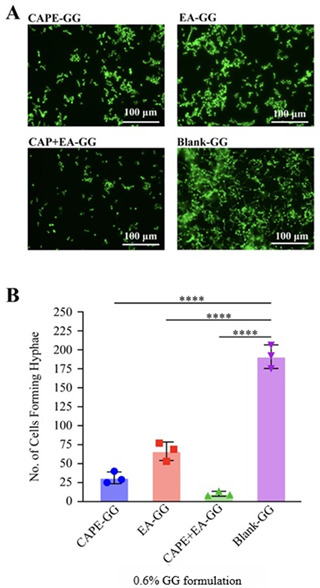
Inhibition of *C. albicans* hyphae formation using the GG formulations loaded with 1,000 µg/mL of CAPE, EA, or CAPE + EA. (A) Representative fluorescence microscopy images of *C. albicans* strain MLR62 tagged with hyphae-specific green fluorescence protein to visualize the inhibition of its hyphal and yeast forms after treatment with the 0.6% GG formulations loaded with CAPE (1,000 µg/mL), EA (1,000 µg/mL), or CAPE (1,000 µg/mL) + EA (1,000 µg/mL). Blank-GG formulation was used as a control. Images showed significantly reduced hyphal structures after treatment with CAPE-GG, EA-GG, and CAPE + EA GG compared with the blank-GG control. Scale bar = 100 µm. (B) Quantification of hyphal inhibition (number of hyphae formed) demonstrated that CAPE-GG, EA-GG, and CAPE-EA-GG exhibited a reduction of *C. albicans* hyphae. The blank-GG formulation had 191 hyphae forming *C. albicans*. Data are presented as mean ± SD (n = 3). Statistical differences were analyzed by one-way analysis of variance. ****P < 0.0001.

Based on the results of our killing kinetics and biofilm disruption experiments, the 0.6% GG formulation (1,000 µg/mL of CAPE, EA, or CAPE + EA) was identified as the most effective concentration. Therefore, the 0.6% GG formulation was used to inhibit *C. albicans* hyphal formation. The CAPE-, EA-, and CAPE + EA-loaded GG formulations all successfully inhibited *C. albicans* hyphal formation within 4 h of treatment. Microscopic analysis revealed that the CAPE + EA GG formulation eliminated hyphal formation by most *C. albicans* cells, whereas the CAPE-GG and EA-GG formulations only moderately inhibited it. In contrast, the control blank-GG allowed extensive and distinct *C. albicans* hyphal formation (Fig. 8A).

Furthermore, the size and morphology of hyphae varied across the treatments. The CAPE-GG and EA-GG formulations resulted in hyphae that were shorter and irregularly sized, whereas the CAPE + EA GG formulation almost eliminated hyphal formation. In comparison, long, even-sized hyphae were observed with the control blank-GG. These findings were corroborated by quantification of the hyphae-forming cells, revealing 31, 63, 10, and 191 hyphae-forming cells following treatment with the CAPE-GG, EA-GG, CAPE + EA GG, and blank-GG formulations, respectively (Fig. 8B).

### Toxicity of the GG formulations against hRBCs and HGF-1 cell lines

**Fig 9:**
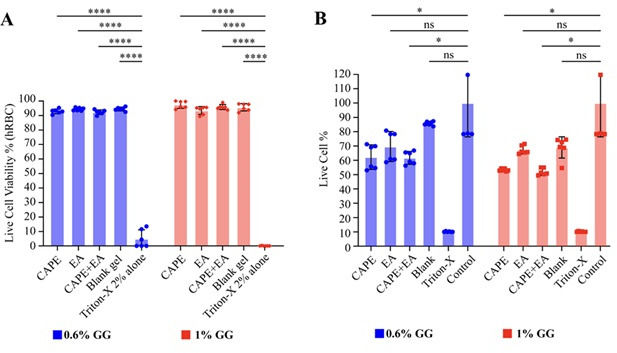
(**A**) Hemolysis assay showing the effects of the 0.6% and 1% gellan gum (GG) formulations loaded with CAPE (1,000 µg/mL), EA (1,000 µg/mL), or CAPE (1,000 µg/mL) + EA (1,000 µg/mL) and blank-GG on live cell viability (%) in 2% hRBCs. Triton X-100 (2%) was used as positive control. Both the 0.6% and 1.0% CAPE-GG, EA-GG, and CAPE + EA GG formulations exhibited high cell viability, with no significant hemolysis compared with positive control. (**B**) WST-1 assay showing the cytotoxicity of the 0.6% and 1.0% GG formulations (CAPE-GG, EA-GG, CAPE + EA GG, and blank-GG) on the HGF-1 cell line. Triton X-100 (2%) served as positive control, and untreated cells were used as negative control. The 0.6% GG formulations showed higher cell viability (62.0%–85.7%) compared with the 1% GG formulations (51%–69%). Triton X-100 demonstrated significant cytotoxicity, reducing cell viability to less than 10%. Data are presented as mean ± SD (*n* = 6). Statistical differences were analyzed by one-way analysis of variance. *P < 0.05, ****P  <  0.0001. ns, non-significant.

To evaluate toxicity, we tested the hemolytic activity of 0.6% and 1.0% GG formulations containing 1,000 µg/mL of CAPE-GG, EA-GG, or CAPE + EA GG against hRBCs. There was no substantial hemolysis resulting from the 0.6% and 1.0% GG formulations (Fig. 9A). The 0.6% GG formulations of CAPE, EA, CAPE + EA and blank-GG exhibited 92.9%, 94.3%, 92.3%, and 94.4% cell viabilities, respectively. The 1% GG formulation of CAPE, EA, CAPE + EA, and blank-GG exhibited 97.5%, 93.4%, 95.8%, and 95.6% cell viabilities, respectively. Triton X (2%) (positive control) showed significantly reduced cell viability, ranging from 0.3% to 4.0%.

We also tested the cytotoxicity of the 0.6% and 1% GG formulations containing 1,000 µg/mL of CAPE-GG, EA-GG, or CAPE + EA GG against HGF-1 cells. The 0.6% GG formulations preserved cell viability, with live cell percentages of 62.0%, 69.0%, 61.7%, and 85.7% for CAPE-GG, EA-GG, CAPE + EA GG, and blank-GG, respectively (Fig. 9B). Similarly, the 1% GG formulations also maintained cell viability, with live cell percentages of 53%, 67%, 51%, and 69%, respectively. However, 2% Triton X-100 (positive control) exhibited high cytotoxicity and reduced cell viability to <10%. These results demonstrate the biocompatibility of GG-based formulations, with a lower concentration of GG (0.6%) offering higher cell viability. The other components of the GG formulation, such as PEG 400 (0.5%), Span 80 (0.5%), and genipin (5 mM), exhibited no toxicity to the HGF-1 cell lines (Fig. S5).

### Drug release and killing of *C. albicans* under artificially simulated chewing of the GG formulations

**Fig 10:**
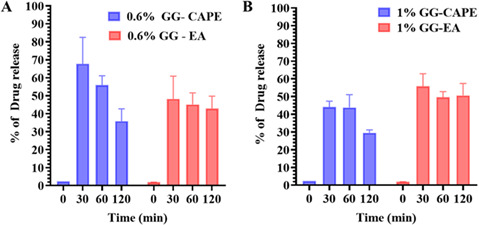
Artificially simulated chewing of the 0.6% and 1.0% GG formulations loaded with CAPE (1,000 µg/mL) and EA (1,000 µg/mL) at 30, 60, and 120. Drug release (%) from the GG formulations at the 30, 60, and 120 min time points. (**A**) For the 0.6% GG formulations, the maximum release of CAPE was 67.7%, whereas EA was 48.2% at 30 min. (**B**) For the 1% GG formulations, the maximum release of CAPE was 44.07%, whereas EA was 55.8% at 60 min. Data are presented as mean ± SD (*n* = 2).

Finally, to simulate drug release from GG formulation, we conducted 30- and 60 min chewing simulations of the 0.6% and 1.0% GG formulations containing CAPE, EA, and CAPE + EA in artificial saliva (Fig. 10A and B). UV-Vis spectra were used to calculate drug release (%). The 0.6% GG formulation yielded a release of 67.7% (30 min), 55.9% (60 min), and 35.8% (120 min) for CAPE and 48.2% (30 min), 45.1% (60 min), and 42.1% (120 min) for EA (Fig. 10A). Likewise, the 1% GG formulation achieved a release of 44.07% (30 min), 43.8% (60 min), and 29.5% (120 min) for CAPE and 55.8% (30 min), 49.6% (60 min), and 50.6% (120 min) for EA (Fig. 10B).

**Fig 11:**
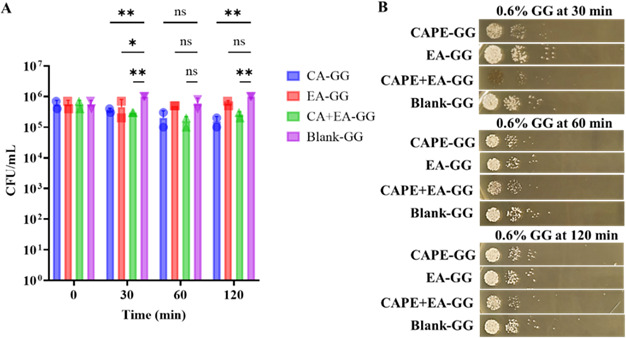
Reduction in *C. albicans* CFU under chewing simulation using 0.6% GG formulations loaded with CAPE (1,000 µg/mL), EA (1,000 µg/mL), or CAPE (1,000 µg/mL) + EA (1,000 µg/mL). (**A**) CFU reduction of *C. albicans* following exposure to 0.6% GG formulations containing CAPE (1,000  µg/mL), EA (1,000  µg/mL), or CAPE + EA (1,000  µg/mL each) at time points 30, 60, and 120  min. At 30  min, CAPE-GG (*P*  =  0.0076), EA-GG (*P*  =  0.0227), and CAPE + EA GG (*P*  =  0.0045) showed significant reductions in CFU compared to the untreated control. At 60 min, none of the formulations showed a significant reduction in CFU. At 120 min, CAPE-GG (*P*  =  0.0011) and CAPE + EA GG (*P*  =  0.0027) again showed significant antifungal effects. (**B**) Comparison of 30–120 min CFU counts highlights the sustained anticandidal efficacy of the 0.6% GG formulations, including CAPE-GG, EA-GG, and CAPE + EA GG, under dynamic chewing simulation. Data are presented as mean ± SD (*n*  =  3). Statistical analysis was performed using two-way analysis of variance.*P < 0.05, **P  <  0.005. ns, not significant.

At 30 min, the 0.6% GG formulation achieved a maximum reduction of 1 log_10_ CFU/mL in *C. albicans* viability. At the 30 min time point, CFUs of *C. albicans* were 3.5 × 10⁵ CFU/mL for CAPE-GG (*P* = 0.0076), 4.5 × 10⁵ CFU/mL for EA-GG (*P* = 0.0227), and 3 × 10⁵ CFU/mL for CAPE + EA GG (*P* = 0.0045) treatment, exhibiting onefold reduction in CFU compared to blank GG (Fig. 11A). At 60 min, no significant reduction was observed in CAPE-GG-treated (*P* = 0.1325), EA-GG-treated (*P* = 0.9993), or CAPE + EA GG-treated (*P* = 0.0730) *C. albicans* compared to blank-GG. At 120 min, compared to blank-GG (4.5 × 10⁶ CFU/mL), CFU counts were significantly reduced in CAPE-GG (1.5 × 10⁵ CFU/mL, *P* = 0.0011) and CAPE + EA GG (2.5 × 10⁵ CFU/mL, *P* = 0.0027), while EA-GG (6 × 10⁵ CFU/mL) showed no significant difference (*P* = 0.1325) (Fig. 11A). These findings were visually corroborated by YPD agar plating, which showed a marked reduction in *C. albicans* colony formation in CAPE-GG, EA-GG, and CAPE + EA GG treatments relative to blank-GG across all time points (Fig. 11B).

**Fig 12:**
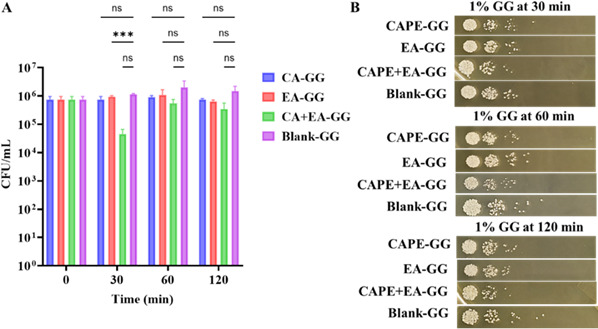
Reduction in *C. albicans* CFU under chewing simulation using 1% GG formulations loaded with CAPE (1,000 µg/mL), EA (1,000 µg/mL), or CAPE (1,000 µg/mL) + EA (1,000 µg/mL). (**A**) CFU reduction of *C. albicans* following exposure to 1% GG formulations containing CAPE (1,000  µg/mL), EA (1,000  µg/mL), or CAPE + EA (1,000  µg/mL each) at time points 30, 60, and 120  min. At 30 min, CAPE-GG (*P*  = 0.452), EA-GG (*P*  < 0.001), and CAPE + EA GG (*P*  =  0.059) showed reductions in CFU compared to the untreated control. At 60 and 120 min, none of the formulations showed significant CFU reduction. (**B**) Comparison of 30–120 min CFU counts highlights the sustained anticandidal efficacy of the 1% GG formulations, including CAPE-GG, EA-GG, and CAPE + EA GG, under dynamic chewing simulation. Data are presented as mean ± SD (*n*  =  3). Statistical analysis was performed using two-way analysis of variance. ***P  <  0.001. ns, not significant.

In the 1% GG formulation, there was a significant reduction in the CFU of *C. albicans* at 30 min (*P* < 0.05). The CFUs of *C. albicans* treated with CAPE-GG, EA-GG, and CAPE + EA GG were 7.5 × 10^5^ CFU/mL (*P* = 0.452), 9.5 × 10^5^ CFU/mL (*P* ≤ 0.001), and 4.5 × 10^4^ CFU/mL (*P* = 0.059), showing 1-2 fold reduction in all treatment groups compared to the Blank GG (1.15 × 10^6^ CFU/mL) respectively (Fig. 12A). After 60 min, no further significant reduction in *C. albicans* CFU was observed. The CFU of *C. albicans* following treatment with CAPE-GG, EA-GG, and CAPE + EA GG were 9 × 10⁵, 1.1 × 10⁵, and 5.5 × 10⁵ CFU/mL, respectively (Fig. 12A). However, the *C. albicans* CFU in blank-GG remained consistently high, indicating (2 × 10^6^ CFU/mL) no inherent antifungal activity in GG vehicles alone. The CFUs of *C. albicans* treated with CAPE-GG, EA-GG, and CAPE + EA GG were 7.5 × 10^5^, 6.5 × 10^5^, and 3.5 × 10^5^ CFU/mL, respectively, at 120 min (Fig. 12A). Similarly, Fig. 12B illustrates the reduction in *C. albicans* CFU counts after 30, 60, and 120 min of treatment with CAPE-GG, EA-GG, and CAPE + EA GG.

## DISCUSSION

OC remains increasingly challenging to treat, especially in cases of persistent colonization or frequent recurrence of *C. albicans* in the oral and pharyngeal regions (49). Prolonged use of conventional antifungal agents can often cause discomfort, breed resistance, and disrupt the oral microbial flora (50). Natural polyphenols, namely, CAPE and EA, known for their anticandidal activity, present promising alternative treatment strategies (23, 51–53). However, their therapeutic application has been limited by issues of viscosity, solubility, and bioavailability (54). To address these limitations, we successfully optimized a GG formulation incorporating CAPE and EA using genipin as a cross-linker, together with GG (0.6% and 1.0%), PEG 400, and Span 80, to enhance solubility and bioavailability. The GG formulation exhibited enhanced drug release and antifungal activity, while all formulations inhibited *C. albicans* growth and biofilm and hyphal formations.

GG-based hydrogels have been widely used as delivery platforms for natural polyphenols such as curcumin and naringenin, with chemical conjugation significantly enhancing their stability and biological activity (55). In addition to natural compounds, GG microparticles have enabled the oral delivery of synthetic drugs, such as methotrexate, by enhancing mucoadhesion and improving bioavailability (56). Previous efforts to encapsulate CAPE or EA as single agents have improved the antifungal efficacy. More specifically, Sampaio et al. (31) demonstrated that cyclodextrin-encapsulated EA exhibited a MIC of 25  µg/mL and inhibited *C. albicans* biofilm formation. Similarly, CAPE-loaded polymeric nanoparticles reduced the MIC of *C. albicans* by twofold (700  µg/mL) compared to free CAPE (1,400  µg/mL), highlighting the role of polymer-based formulation in enhancing antifungal efficacy (57). A recent study developed an oral GG hydrogel encapsulating CAPE for antifungal applications against *C. albicans*, demonstrating promising *in vitro* antifungal activity (22).

Even though CAPE (12, 58) or EA (12) individually exhibit antifungal activity against the planktonic, biofilm, and hyphal forms of *C. albicans*, mechanistically, caffeic acid derivatives disrupt the fungal cell wall by inhibiting β-1,3-glucan synthase (59) and synergize with caspofungin by disrupting fungal iron homeostasis (60). EA, on the other hand, induces cell wall stress, which is evidenced by an increase in MIC values in the presence of osmotic stabilizers (12). Additionally, exposure to EA results in the inhibition of *Candida* virulence factors, such as phospholipases (12). The combined use of CAPE and EA within a single polymeric delivery system has not been explored. Using a checkerboard assay, we confirmed the synergistic interaction between CAPE and EA, yielding a FICI below 0.5. Because of these findings and the ability of polyphenols such as CAPE and EA to form non-covalent interactions with polysaccharides like GG, primarily through hydrogen bonding and hydrophobic interactions (61), we formed the hypothesis that a combined hydrogel system could enhance compound stabilization and enable controlled release.

Interestingly, in our foundational studies, we found that genipin enhanced drug stability and release in GG formulations. Genipin is a natural cross-linker with low cytotoxicity, efficiently stabilizes biomacromolecules (62), and unlike CaCl_2_, which may interfere with antifungal efficacy by inhibiting salivary histatin 5 (63), genipin is associated with controlled CAPE and EA release and improves antifungal activity. Other cross-linkers like polyvinyl alcohol (64), dimethyl fumarate (65), or magnesium ions (Mg²^+^) (66) have been explored, but genipin offers optimal structural integrity and sustained drug delivery, aligning with previous findings (67).

We confirmed that the formulation containing GG, genipin, PEG 400, and Span 80 showed the absorbance peak for CAPE and EA at 325 and 255 nm, respectively. The incorporation of PEG 400 (68) and Span 80 (69, 70) enhanced solubility, bioavailability, and better drug dispersion of CAPE and EA. Furthermore, the drug-release rate determines the efficacy of GG formulation. These findings align with previous studies, demonstrating characteristic absorption peaks that confirm the successful incorporation and release of these polyphenols. Garcia et al. (22) formulated CAPE-loaded GG with CaCl_2_ and identified absorption peaks at 260 nm for GG and 325 nm for CAPE using UV-Vis spectroscopy. Similarly, Li et al. characterized EA release from an ellagic acid solid dispersion prepared with polyvinylpyrrolidone, observing distinct absorption peaks at 255 and 360 nm (71). Our results further validate these findings, showing similar absorption peaks at 325 nm for CAPE and 255/360 nm for EA, confirming their successful release from the GG formulation. This consistency across studies underscores the reliability of UV-Vis analysis in quantifying drug release and highlights the potential of GG as an effective carrier for sustained CAPE and EA delivery in antifungal applications.

The killing kinetics assays demonstrated differences in the rate of *C. albicans* eradication by CAPE-GG, EA-GG, and CAPE + EA GG under static versus shaking conditions. Shaking incubation notably increased the release of CAPE, EA, and CAPE + EA, exhibiting faster reduction in *C. albicans* CFU counts within 30 min of treatment. In contrast, static incubation delayed CAPE and EA release, with a notable decrease in CFU observed after 60 min. Our results align with those of Garcia et al. (22), who reported that CAPE-GG formulations under static conditions reduced *C. albicans* viability after 2 and 12 h of treatment. Notably, the 1.0% GG formulation exhibited superior antifungal efficacy compared to the 0.6% formulation, likely due to increased hydrogel density that enhances drug retention and maintains higher local drug concentrations at the site of infection (72).

Under static conditions (Fig. 5), however, the 1% CAPE + EA GG formulation showed reduced antifungal activity, possibly due to restricted drug diffusion from the denser gellan matrix. Increased gellan concentration leads to higher gel rigidity and reduced matrix porosity (73), limiting passive diffusion of encapsulated agents. In contrast, shaking conditions (Fig. 6) promote mechanical erosion and facilitate drug release, resulting in more effective *C. albicans* killing (74). The mechanical stability of the denser gel also prolongs drug–fungus contact, particularly within biofilms and hyphal networks (22). Antifungal efficacy, therefore, depends not only on the quantity of drug released but also on the dynamics and mechanism of release. Overall, the findings highlight the critical role of external mechanical forces, such as agitation or chewing-induced shear stress, in enhancing drug diffusion from hydrogels and support their potential application in dynamic oral environments.

*C. albicans* exhibits dimorphic growth, transitioning between yeast and filamentous forms, a key virulence trait that facilitates both planktonic proliferation and biofilm development (75, 76). Biofilm formation is a key factor in mucosal and systemic infections and is often resistant to treatment due to its complex structure of yeast cells, pseudohyphae, hyphae, and extracellular matrix (77). CAPE and EA both have inhibitory and disruptive effects on biofilm (22, 53) and CAPE inhibits hyphal development at a concentration of 8 µg/mL and suppresses the yeast-to-hyphae transition at 16 µg/mL (12). We found that the CAPE +EA GG (1,000 + 1,000 µg/mL) formulation effectively disrupted biofilms within 4 h and outperformed the individual molecules by sustaining gradual drug release and enhancing therapeutic efficacy.

Cytotoxicity assays using HGF-1 cells demonstrated that the CAPE and EA formulations were biocompatible. To date, few studies have directly assessed the oral mucosal toxicity of CAPE and EA. Kurek-Górecka et al. (78) reported that CAPE did not exert cytotoxic effects on HGF-1 cells and inhibited the production of tumor necrosis factor alpha and interleukin-6 in HGF-1 cells stimulated with LPS and interferon alpha. Another report indicated that CAPE exhibits minimal cytotoxicity toward keratinocytes at concentrations up to 160 µg/mL, whereas EA demonstrates slight cytotoxicity in its non-complexed form (23). However, no studies have specifically evaluated the cytotoxic effects of EA on HGF-1 cells. Additional experimentation is needed to determine whether a balance can be struck between antifungal efficacy and minimized cytotoxicity.

There is a significant gap in existing literature regarding the application of artificial chewing simulations to study both drug-release kinetics and the antifungal effects of CAPE and EA. Our study helps to address this gap, providing novel insights into the combined effects of mechanical forces and drug diffusion on antifungal activity. To our knowledge, the antifungal efficacy of GG polyphenol systems under simulated masticatory conditions, which more closely mimic the oral environment, had not been studied prior to this work and should be further explored in future studies. We used a compression system to simulate the chewing mechanism and assess drug release from the formulation under dynamic conditions. The artificial chewing simulation enables the quantification of CAPE and EA release from the GG formulations at defined time intervals, in this case, every 30 min. Furthermore, the artificial chewing simulation facilitates the evaluation of *C. albicans* CFU reductions under chewing motion that offers a complete understanding of the antifungal efficacy of the formulation.

The observed decline in drug-release rate during the artificial chewing simulations can be attributed to multiple interrelated factors. Initially, drug release occurs rapidly due to the cleavage of more accessible linkers and the diffusion of drug molecules near the hydrogel surface (79). The “initial burst” phase is driven by steeper concentration gradients and shorter diffusion paths (79). As time progresses, drug molecules more deeply embedded within the hydrogel network require longer diffusion paths and face reduced concentration gradients, significantly slowing their transport to the surface (80). The slowing of the drug release leads to an exponential decay in efflux over time, wherein the percentage of drug released per unit of time naturally declines (80). Furthermore, the network architecture of the hydrogel is critical in modulating this release behavior. Increased cross-linker density creates a tighter network that can restrict the mobility of drug molecules and hinder further degradation or diffusion, especially at later stages of incubation (81). This structural constraint can trap residual drug within the matrix, contributing to the apparent plateau or slower release at prolonged time points (81).

However, the artificial chewing simulations do not replace *in vivo* studies. The primary limitation of the Flexcell system is that, although it induces mechanical shear, it fails to replicate salivary clearance, host immune responses, and the complexity of the oral microbiota. The *in vitro* chewing model using Flexcell does not fully replicate the oral environment because it lacks key components of human saliva, such as histatins (82) and defensins (83), which influence antifungal activity (84). Additionally, unlike the in *vivo* setting, the established assay does not simulate continuous salivary flow or swallowing, which dilutes polyphenols and removes planktonic yeast, reducing their contact time.

### Conclusion

We evaluated GG-based formulations incorporating CAPE, EA, or CAPE + EA as an effective treatment for OC and identified two optimized formulations containing 0.6% and 1.0% GG that were effective in terms of antifungal efficacy, drug release, and biocompatibility. The CAPE + EA GG formulation effectively reduced *C. albicans* viability, disrupted biofilm formation, and inhibited hyphal development that addresses the key challenges such as antifungal resistance, recurrence, and adverse effects of conventional therapies. Compared with the 1.0% GG formulation, the 0.6% GG formulation exhibited faster drug release while maintaining significant antifungal activity. Using genipin as a cross-linker facilitated sustained drug release and antifungal activity while maintaining safety, while PEG 400 and Span 80 improved solubility, stability, and bioavailability. Additionally, artificial chewing simulations displayed the controlled drug release and killing of *C. albicans*. Our study is the first to evaluate CAPE and EA co-loaded GG formulations against *C. albicans* under simulated chewing conditions, laying the groundwork for further optimization and *in vivo* validation to enhance drug-release kinetics and antifungal potency for oral therapeutic applications.

## SUPPLEMENTAL MATERIAL

Supplemental material spectrum.00798-25-s0001.docx Fig. S1 to S5; Supplemental methods.

ASM does not own the copyrights to Supplemental Material that may be linked to, or accessed through, an article. The authors have granted ASM a non-exclusive, world-wide license to publish the Supplemental Material files. Please contact the corresponding author directly for reuse.
