# Supplementary material for: Gellan gum formulations containing natural polyphenolic compounds to treat oral candidiasis
Source: Microbiol Spectr. 2025 Aug 6;13(9):e00798-25. doi: 10.1128/spectrum.00798-25 (PMC12403768; doi:10.1128/spectrum.00798-25)
Supplement: Supplemental material — Fig. S1 to S5; Supplemental methods. [file spectrum.00798-25-s0001.docx]

**Supplementary Methods**

**Checkerboard Assay for Evaluating CAPE and EA Interaction**

We prepared 10× stock solutions of CAPE (160 μg/mL) and EA (80 μg/mL), which were at least threefold higher than their respective MIC values. We added 10 μL of each stock solution to a 96-well plate in an 8 × 8 checkerboard format, with CAPE diluted along the Y-axis and EA along the X-axis. Next, we added 90 μL of a standardized *C. albicans* suspension (1 × 10³ CFU/mL) to each well containing the drug combinations. We incubated the plates at 30 °C for 24 hrs under aerobic conditions, following the protocol described by Bhattacharya *et al*. (1). All experiments were performed in duplicate. After incubation, we measured the OD at 600 nm using a SpectraMax M2 Multi-Mode Microplate Reader (Molecular Devices, CA, USA) to assess fungal growth inhibition. We evaluated the interaction between CAPE and EA by calculating the sum of fractional inhibitory concentrations (ΣFIC) for each drug combination using the formula:

ΣFIC = MIC of CAPE in combination/MIC of CAPE alone + MIC of EA in combination/MIC of EA alone

A ΣFIC ≤ 0.5 indicates synergy, 0.5 < ΣFIC ≤ 1 indicates additivity, 1 < ΣFIC ≤ 4 indicates indifference, and ΣFIC > 4 indicates antagonism (2).

**Supplementary Figures**

**Fig S1.** The heatmap illustrates the results of a checkerboard microdilution assay performed to assess the interaction between CAPE and EA against *C. albicans*. CAPE was diluted along the Y-axis and EA along the X-axis in a two-dimensional matrix. The FIC index was calculated for each drug combination. FIC values ≤ 0.5 indicate synergistic interactions, as shown by lighter regions on the heatmap. Strong synergy was observed at FIC = 0.25 of CAPE and EA.


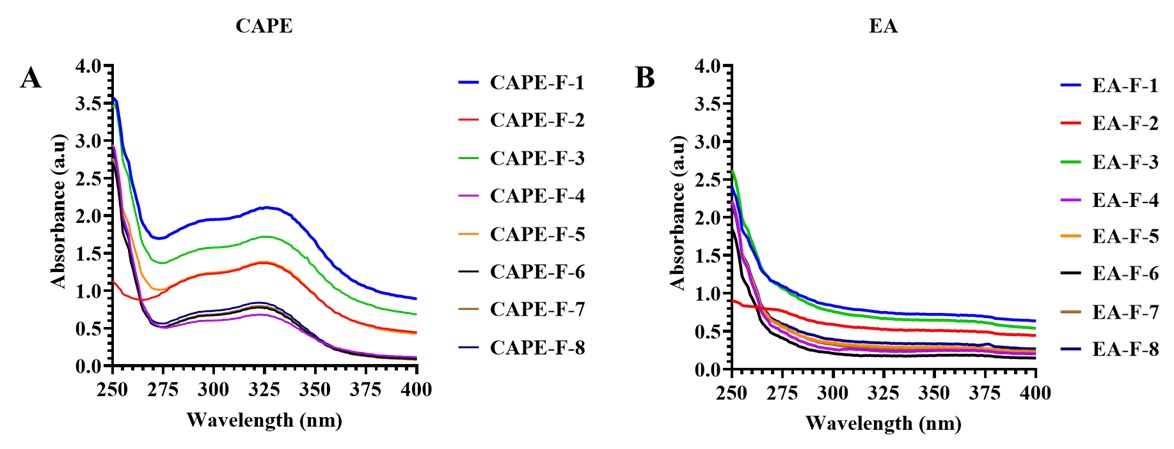


**Supplementary Figure 2.** UV-vis spectrum of CAPE (1000 μg/mL) and EA (1000 μg/mL) released from different chemical combinations of GG formulation at 60 min was measured at 250- 400 nm for every 1 nm wavelength. (A) The release of CAPE showed peak formation at 325 nm. (B) The release of EA showed peak formation at 325 nm.

*
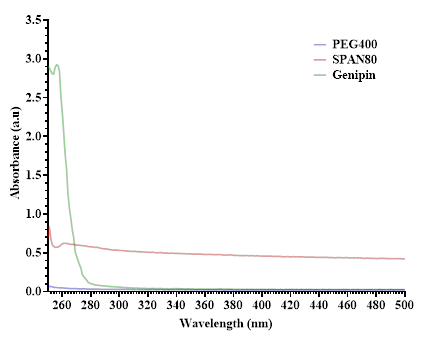
*

**Fig. S3.** UV-Vis spectra of PEG 400 (0.5%), Span 80 (0.5%), and genipin (5 mM) were recorded over a wavelength range of 250–500 nm. These individual components of the GG formulation showed no significant spectral interference with CAPE- or EA-loaded GG formulations*.*


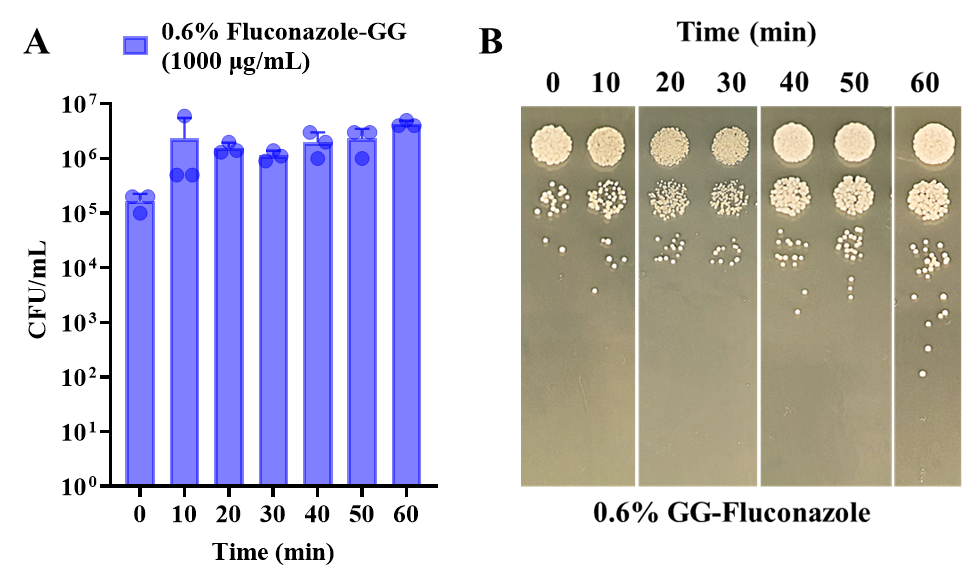


**Fig S4.** Killing kinetics of *C. albicans* treated with the 0.6% GG formulation loaded Fluconazole (1000 μg/mL) over 60 min.(A) Quantitative analysis of the anticandidal activity of the Fluconazole loaded 0.6% GG formulations showed no significant reductions in viable fungal colonies. **B)** The visual representation of *C. albicans* colonies demonstrated an increase in CFUs as the duration of treatment increased from 0 to 60 min (n = 3; mean ± SD).

**Fig. S5.** WST-1 assay showing the cytotoxicity of PEG400 (0.5%), SPAN80 (0.5%), Genipin (5mM), and Blank-GG on HGF-1 cell line.

**References**

1. Bhattacharya R, Sharma P, Bose D, Singh M. 2024. Synergistic potential of α-Phellandrene combined with conventional antifungal agents and its mechanism against antibiotic resistant Candida albicans. CABI Agriculture and Bioscience 5:17.

2. Garcia LS. 2010. Clinical microbiology procedures handbook, vol 1. American Society for Microbiology Press.
